# High-Throughput Sequencing-Based Identification of Serum Exosomal Differential miRNAs in High-Grade Glioma and Intracranial Lymphoma

**DOI:** 10.1155/2020/2102645

**Published:** 2020-10-07

**Authors:** Shun Wang, Zhenkuan Xu, Chao Zhang, Rui Yu, Jun Jiang, Chengwei Wang, Chuncheng Qu

**Affiliations:** ^1^Department of Clinical Laboratory, The Second Hospital, Cheeloo College of Medicine, Shandong University, Jinan, 250012 Shandong, China; ^2^Department of Neurosurgery, The Second Hospital, Cheeloo College of Medicine, Shandong University, Jinan, Shandong 250033, China; ^3^Department of Clinical Laboratory, The Ninth People's Hospital of Qingdao, Qingdao, Shandong 266000, China

## Abstract

**Objective:**

At present, no effective noninvasive method is currently available for the differential diagnosis of high-grade glioma and intracranial lymphoma. In the present study, we aimed to screen microRNA (miRNA) markers in serum exosomes for differential diagnosis of high-grade glioma and intracranial lymphoma using high-throughput sequencing technology.

**Methods:**

Patients with intracranial lymphoma or high-grade glioma and healthy controls were included in this study (training cohort (*n* = 10) and validation cohort: intracranial lymphoma (*n* = 10), high-grade glioma (*n* = 32), and healthy controls (*n* = 20)). After RNA was extracted from serum exosomes, the high-throughput sequencing was used to determine the expression profiles of serum exosomal miRNAs and screen the differentially expressed miRNAs. RT-qPCR was used to verify the expressions of the selected miRNAs. The differences of miRNA expressions between groups were assessed by the Kruskal-Wallis test. The diagnostic value was analyzed using the receiver operating characteristic (ROC) curve.

**Results:**

High-throughput sequencing demonstrated that 170 miRNAs, including 109 upregulated ones and 61 downregulated ones, were differentially expressed in serum exosomes between the patients with intracranial lymphoma and high-grade glioma. Compared with the healthy controls, the number of differential serum exosomal miRNAs in the high-grade glioma group and intracranial lymphoma group was 130 and 173, respectively. RT-qPCR proved that both miR-766-5p and miR-376b-5p were significantly downregulated in high-grade glioma and intracranial lymphoma patients compared with the healthy controls (all *p* < 0.001), and the expression of serum exosomal miR-766-5p in the intracranial lymphoma group was lower compared with the high-grade glioma group (*p* < 0.05). The areas under ROC curve (AUCs) of serum exosomal miR-766-5p and miR-376b-5p for the diagnosis of glioma were 0.8883 (*p* < 0.001) and 0.7688 (*p* = 0.001), respectively, and they were 0.9271 (*p* < 0.001) and 0.8542 (*p* < 0.001), respectively, for the diagnosis of intracranial lymphoma. Moreover, the AUC value of serum exosomal miR-766-5p for the differential diagnosis of glioma and intracranial lymphoma was 0.7201 (*p* = 0.026).

**Conclusions:**

miR-766-5p and miR-376b-5p in serum exosomes might be used as auxiliary diagnostic indicators for high-grade glioma and intracranial lymphoma, and miR-766-5p might be used as a differential diagnostic marker for both diseases.

## 1. Introduction

High-grade glioma and intracranial lymphoma are all highly proliferative and aggressive tumors with a poor prognosis and short survival. It is difficult or even impossible to distinguish high-grade glioma and intracranial lymphoma due to the similarity of their appearance on the conventional CT and MRI examinations [1]. Biopsy remains the gold standard to distinguish intracranial lymphoma and high-grade glioma, while many patients cannot accept such invasive examination approach. However, it is very necessary to differentiate them, because the therapeutic methods of them are quite different. Intracranial lymphoma is more sensitive to chemotherapy and radiation [2]. Therefore, it is urgently necessary to identify an efficient and noninvasive detection indicator for the differential diagnosis of both diseases.

MicroRNA (miRNA) is an endogenous noncoding small molecule RNA of approximately 19-25 nucleotides in length in eukaryotes [3]. It is involved in many important biological processes, such as cell growth, differentiation, proliferation, and apoptosis [4–6]. Recently, abnormal expressions of miRNAs have been found in several malignant tumors. A variety of miRNAs have been shown to play important roles in the occurrence and development of glioma and intracranial lymphoma [7–10]. Moreover, they have potential application values for diagnosis and prognostication of these two fatal diseases [10, 11].

Exosomes are vesicles secreted by cells with double-layered lipid membranes, which are important tools for the exchange of living activities between cells [12]. Due to their small particle size and strong permeability in the body, glioma cell-derived exosomes can be detected even in the serum of some glioma patients whose blood-brain barrier remains intact [13]. Therefore, miRNAs in peripheral blood exosomes can be potential markers and therapeutic targets for neurological tumors. In the present study, we evaluated the expression profiles of serum exosomal miRNAs in patients with intracranial lymphoma, high-grade glioma, and healthy controls by high-throughput sequencing. The differential miRNAs were screened between various groups. Moreover, we further verified that miR-766-5p and miR-376b-5p in serum exosomes were significantly downregulated in both diseases, and miR-766-5p could be used as a differential diagnostic marker for intracranial lymphoma and high-grade glioma.

## 2. Materials and Methods

### 2.1. Serum Specimens and Clinical Characteristics

This study was approved by the Clinical Research Ethics Committee of the Second Hospital, Cheeloo College of Medicine, Shandong University, and written informed consent was obtained from each participant. A total of 94 subjects who received treatment in the Second Hospital and Qilu Hospital of Cheeloo College of Medicine from June 2015 to May 2017 were enrolled in this study, including 22 patients with intracranial lymphoma, 42 patients with high-grade glioma, and 30 healthy controls with no history of malignant tumor and no abnormal indicators of laboratory tests. The diagnosis of all patients was confirmed by craniotomy and intraoperative and postoperative pathology. All patients did not undergo chemotherapy or radiotherapy before surgery. The pathology of patients was examined according to WHO Classification of Tumors of the Central Nervous System, which was completed by two clinical pathologists at Shandong University Second Hospital and Qilu Hospital. The glioma patients selected in this study were all WHO III and IV patients. Demographic and clinical characteristics are described in Table 1. Venous blood (5 mL) under fasting conditions was collected from all patients and normal controls in the early morning, followed by centrifugation at 3,500 rpm for 10 min at 4°C within 2 h after collection. Samples with hemolysis or lipemia were removed. The upper serum was collected and centrifuged again at 12,000 rpm for 10 min to remove blood cell debris. The upper serum was placed in RNase-free EP tubes and stored at -80°C.

### 2.2. Extraction of Serum Exosomes

Serum exosomes were isolated using the ExoQuick™ Exosome Precipitation Solution Kit (System Biosciences, CA, USA) according to the manufacturer's instructions. Briefly, the serum was centrifuged at 3,000 *g* for 15 min, and then 63 *μ*L ExoQuick precipitation solution was added to 250 *μ*L serum, followed by sufficient mixing and incubation for 30 min. The mixture was then centrifuged at 1,500 *g* for 30 min (4°C). The supernatant was discarded, and the pellet was centrifuged again. Subsequently, the pellet was resuspended in 250 *μ*L PBS. Particle Metrix ZetaView was used to detect extracellular vesicle [14].

### 2.3. RNA Extraction from Serum-Derived Exosomes

Total RNA was extracted using the RNeasy Micro Kit (QIAGEN, Germany) according to the manufacturer's instructions. The sample was mixed with 700 *μ*L Qiazol Lysis Reagent, followed by incubation at room temperature for 5 min, and then 140 *μ*L chloroform was added, followed by incubation at room temperature for 2 min. The mixture was centrifuged at 1,200 *g* for 15 min at 4°C. The supernatant was collected, followed by addition of 1.5 volumes of absolute ethanol. After mixing, 700 *μ*L sample was transferred into a spin column and centrifuged at 12,000 *g* for 15 s at room temperature. The filtrate was discarded, and 700 *μ*L Buffer RWT was added onto the spin column, followed by centrifugation at 12,000 *g* for 15 s at room temperature. The filtrate was discarded, and 500 *μ*L Buffer RPE was added, followed by centrifugation at 12,000 *g* for 15 s at room temperature. The filtrate was discarded again, and 500 *μ*L 80% ethanol was added, followed by centrifugation at 12,000 *g* for 15 s at room temperature. The spin column was placed into a new 2 mL collection tube and centrifuged at 13,300 *g* for 5 min at room temperature. The spin column was placed into a new collection tube again, and 16 *μ*L RNase-free H_2_O was added. The spin column was subjected to centrifugation 13,300 *g* for 1 min at room temperature, and the filtrate was collected.

### 2.4. High-Throughput Sequencing

Ten cases were randomly selected from each group, the serum samples of each group were divided into three parts (*n* = 3, *n* = 3, and *n* = 4) and mixed, and exosomes were isolated. After extraction of the exosomal RNA, the high-throughput sequencing was performed by Novogene Biotechnology Co., Ltd. (Beijing, China). The procedures of total RNA detection, gene library construction, and HiSeq/MiSeq sequencing were carried out according to the manufacturer's instructions. Small RNA Sample Prep Kit was used for gene library construction. Agilent 2100 was adopted to detect the insert size of the library. After the detection results met the expectations, quantitative polymerase chain reaction (qPCR) was employed to quantify the effective concentration of the library (effective concentration > 2 nM) to ensure the quality of the library.

### 2.5. qPCR for miRNAs

The primers were synthesized by GenePharma Co., Ltd. (Shanghai, China). The primer sequences were as follows: miR-766-5p-forward: TTGCTAGGAGGAGGAATTGGTG, miR-766-5p-reverse: TATGGTTGTTCACGACTGGTTCAC; miR-376b-5p-forward: CACAGCCGTGGATATTCCTTCT, miR-376b-5p-reverse: GTGCAGGGTCCGAGGT; and U6-forward: CAGCACATATACTAAAATTGGAACG, U6-reverse: ACGAATTTGCGTGTCATCC. TB Green® Premix Ex Taq™ (Tli RNaseH Plus) Kit (Takara, Tokyo, Japan) was used for qPCR according to the manufacturer's instructions.

### 2.6. Statistical Analysis

All data were analyzed by SPSS 20.0 statistical software. The Kruskal-Wallis test was used to determine the difference of miRNA expressions among high-grade glioma, intracranial lymphoma, and normal controls. The Mann-Whitney test was used to assess the difference of miRNA expressions between high-grade glioma and intracranial lymphoma, between high-grade glioma and normal controls, or between intracranial lymphoma and normal controls. Receiver operating characteristic (ROC) curve was used to analyze the diagnostic value. *p* < 0.05 was considered statistically significant.

## 3. Results

### 3.1. Characterization of Exosomes Isolated from Serum in Three Groups

**Figure 1:**
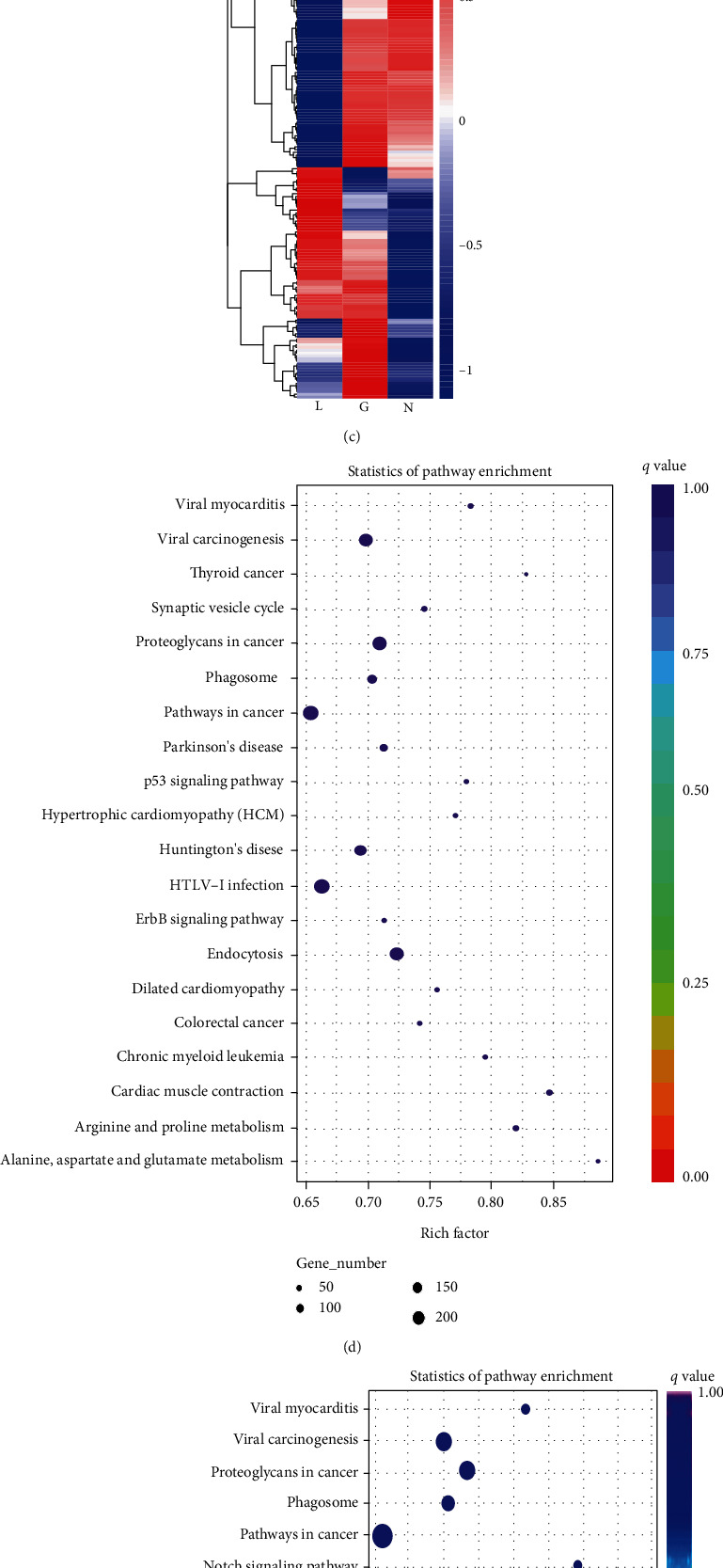
Characterization of exosomes isolated from serum in three groups. (a) Volcano map of the differential miRNAs of lymphoma (L) and glioma (G). The abscissa represents the fold change of miRNA expression, the ordinate represents the significant degree of change in the expression of miRNA, the scattered dots in the figure represent each miRNA, the blue dots represent miRNAs without significant differences, and the red dots represent significantly upregulated differential miRNAs. The green dots represent significantly downregulated differential miRNAs. *N*_(L)_ = 10, *N*_(G)_ = 10, *N*_(N)_ = 10. (b) Venn diagram of differential miRNAs in intracranial lymphoma (L), high-grade glioma (G), and normal control (N). (c) The clustering map of differential miRNAs in intracranial lymphoma (L), high-grade glioma (G), and normal control (N). Clustering was performed with log_10_ (TPM + 1) values. Red represents high-expressing miRNAs, and blue represents low-expressing miRNAs. (d) The KEGG pathway enrichment analysis for target genes of differentially expressed miRNAs in serum exosomes of glioma. (e) The KEGG pathway enrichment analysis for target genes of differentially expressed miRNAs in serum exosomes of intracranial lymphoma.

The sequencing identified 513 known miRNA mature bodies and one new miRNA mature body of serum exosomes in the high-grade glioma group, while serum exosomes of the intracranial lymphoma group contained 529 known miRNA mature bodies and two new miRNA mature bodies. Moreover, 576 known miRNA mature bodies and seven new miRNA mature bodies were found in serum exosomes of normal controls. Figures 1(a) and 1(b) show that the 170 miRNAs, including 109 upregulated ones and 61 downregulated ones, were differentially expressed in serum exosomes between the intracranial lymphoma group and high-grade glioma group. Compared with the normal controls, there were 130 differential miRNAs in the high-grade glioma group and 173 differential miRNAs in the intracranial lymphoma group. miR-122-5p, miR-1301-3p, miR-194-5p, miR-370-3p, miR-328-3p, miR-181a-5p, miR-191-5p, miR-328-3p, miR-133a-3p, miR-494-3p, miR-331-5p, miR-532-5p, miR-370-3p, let-7c-5p, miR-766-5p, and so on were differentially expressed in serum exosomes of the high-grade glioma group compared with the intracranial lymphoma group, and they were all differentially expressed in serum exosomes of the high-grade glioma group and intracranial lymphoma group compared with the normal controls.

According to the correspondence between miRNA and its target genes, the KEGG (Kyoto Encyclopedia of Genes and Genomes) pathway enrichment analysis listed 20 significantly enriched pathways involved in the regulation of glioma and intracranial lymphoma (Figures 1(d) and 1(e)). Among these pathways, there were many pathways related to physiological and pathological angiogenesis, including focal adhesion, proteoglycans in cancer, and pathways in cancer.

### 3.2. Expression of miRNAs from Serum Exosomes of Patients with Intracranial Lymphoma and High-Grade Glioma

**Figure 2:**
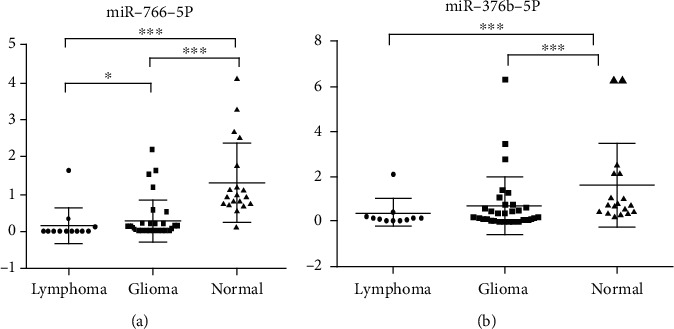
Expressions of miRNAs from serum exosomes of patients with intracranial lymphoma and high-grade glioma. RT-qPCR was used to verify the expressions of the selected miRNAs in serum exosomes. (a) The miR-766-5p expression in three groups. (b) The miR-376b-5p expression in three groups. *N*_(L)_ = 12, *N*_(G)_ = 32, *N*_(N)_ = 20. Data were shown as the mean ± SD. ^∗^*p* < 0.05, ^∗∗^*p* < 0.01, and ^∗∗∗^*p* < 0.001. L: intracranial lymphoma; G: high-grade glioma; N: normal control.

The main clinical problem is that some patients with intracranial lymphoma are mistakenly treated as glioma and given craniotomy. We selected miR-370-3p, miR-328-3p, miR-766-5p, and miR-376b-5p, which were differentially expressed between the high-grade glioma and intracranial lymphoma groups, and verified their differential expressions in the serum exosomes of 12 patients with intracranial lymphoma, 32 patients with high-grade glioma, and 20 healthy controls. Our verification started with glioma patients. The expressions of serum exosomal miR-370-3p and miR-328-3p in high-grade glioma patients were not significantly different compared with the healthy controls (1.18 vs. 0.81, *p* = 0.27; 0.75 vs. 1.06, *p* = 0.23), while we did not verify them in the intracranial lymphoma group. Moreover, the expressions of serum exosomal miR-766-5p and miR-376b-5p were significantly downregulated in both intracranial lymphoma and high-grade glioma patients compared with the healthy controls (miR-766-5p: 0.0048, 0.078 vs. 0.94; miR-376b-5p: 0.16, 0.20 vs. 0.80; all *p* < 0.001) (Figures 2(a) and 2(b)). In the comparison of intracranial lymphoma and high-grade glioma, the data showed that the miR-766-5p expression in serum exosomes of intracranial lymphoma patients was obviously lower compared with the high-grade glioma patients (*p* = 0.03, Figure 2(a)). These results indicated that miR-766-5p and miR-376b-5p in serum exosomes might be diagnostic indicators for high-grade glioma and intracranial lymphoma, and serum exosomal miR-766-5p could be used as a potentially differential diagnostic marker for both diseases.

There was a significant correlation between the expression of serum exosomal miR-766-5p and glioma histological grade (III, IV) (*r* = −0.7427, *p* < 0.001). In contrast, the expression of serum exosomal miR-376b-5p was not related to glioma histological grade (*p* = 0.33).

### 3.3. Diagnostic Efficacy Analysis of miR-766-5p and miR-376b-5p for Intracranial Lymphoma and High-Grade Glioma

**Figure 3:**
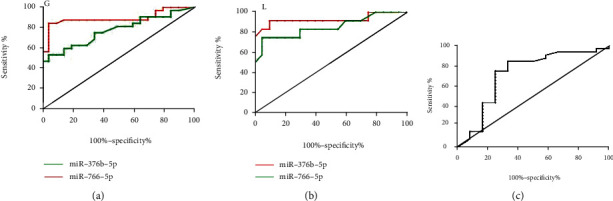
Diagnostic efficacy analysis of miR-766-5p and miR-376b-5p for intracranial lymphoma and high-grade glioma. (a) The AUCs of miR-766-5p and miR-376b-5p for diagnosis of high-grade glioma (G). (b) The AUCs of miR-766-5p and miR-376b-5p for the diagnosis of intracranial lymphoma (L). (c) The AUC value of miR-766-5p in the differential diagnosis for high-grade glioma and intracranial lymphoma. AUCs: areas under ROC curve.

The areas under ROC curve (AUCs) of serum exosomal miR-766-5p and miR-376b-5p for high-grade glioma diagnosis were 0.8883 (*p* < 0.001) and 0.7688 (*p* = 0.001), respectively (Figure 3(a)). The AUCs of serum exosomal miR-766-5p and miR-376b-5p for the diagnosis of intracranial lymphoma were 0.9271 (*p* < 0.001) and 0.8542 (*p* < 0.001), respectively (Figure 3(b)). The AUC of serum exosomal miR-766-5p in the differential diagnosis for high-grade glioma and intracranial lymphoma was 0.7201 (*p* = 0.026) (Figure 3(c)). These data suggested that the expressions of miR-766-5p and miR-376b-5p in serum exosomes could be used for auxiliary diagnosis of high-grade glioma and intracranial lymphoma, while miR-766-5p had a higher diagnostic efficacy. Moreover, serum exosomal miR-766-5p could also be used as a differential diagnostic marker for high-grade glioma and intracranial lymphoma.

### 3.4. Correlation Analysis between miR-766-5p and miR-376b-5p and Clinical Characteristics

Among 32 cases of high-grade glioma, 15 cases were of WHO III classification, and 17 cases were of WHO IV classification. Correlation analysis showed that the expression of serum exosomal miR-766-5p was negatively correlated with WHO grade of glioma (*p* < 0.001, *r* = −0.7427). There was no correlation between the expression of serum exosomal miR-376b-5p and WHO classification of glioma (*p* = 0.33). In addition, there was no correlation between the expression of serum exosomal miR-766-5p/miR-376b-5p and Ki-67 scores (*p* = 0.47, *p* = 0.09). These data indicated that the expression of serum exosomal miR-766-5p might serve as a detection index for glioma classification, although more clinical trials are required to verify such hypothesis.

## 4. Discussion

To date, miRNAs related to the differential diagnosis of high-grade glioma and intracranial lymphoma have not been systematically studied. High-throughput sequencing technology possesses high throughput and high accuracy. Compared with chip sequencing, new miRNA molecules can be obtained [15]. In the present study, differential expression profiles of high-grade glioma and intracranial lymphoma were successfully constructed via using high-throughput sequencing technology.

A large number of studies on miRNAs have shown that targeted therapy based on miRNAs to inhibit or restore expression may be a promising anticancer treatment. Our study screened out two miRNAs in serum exosomes, which were related to the diagnosis of high-grade glioma and intracranial lymphoma. A variety of studies on miR-766 have focused on tumor-related diseases. The miR-766-5p expression is negatively regulated by PRKCZ-AS1 gene in lung adenocarcinoma cells, resulting the promotion of non-small-cell lung cancer [16]. Bioinformatics analysis has shown that salivary miR-766 is a marker to diagnose colorectal cancer [17]. Overexpression of circular miR-766 inhibits the expression of metastasis-associated protein 3, leading to carcinogenesis in hepatocellular carcinoma [18]. In our study, we found that serum exosomal miR-766-5p was significantly downregulated in high-grade glioma and intracranial lymphoma, indicating that miR-766-5p could be used as a diagnostic marker. Mao et al. [19] have reported that inhibition of miR-766-5p leads to the deterioration of glioma by MIR4697HG/miR-766-5p/PRR12 axis. This result is consistent with our data. miR-766-5p in serum exosomes might be a potential diagnostic marker for glioma, especially high-grade glioma. Furthermore, we also demonstrated that its expression was markedly reduced in intracranial lymphoma, which has not been reported. The downregulation of miR-766-5p in intracranial lymphoma was greater compared with the high-grade glioma, indicating that miR-766-5p in serum exosomes could be a differential diagnostic marker for both diseases. This conclusion needs to be validated by clinical trials with larger sample size.

In malignant tumor-related diseases, MEG3 and miR-376b are downregulated in patients with clinically nonfunctioning pituitary adenomas. Upregulation of MEG3 and miR-376b suppresses tumorigenesis and promotes apoptosis of pituitary-derived folliculostellate cells [20]. In patients with metastatic renal cell carcinoma, miR-376b has been demonstrated to be differentially expressed in patients with primary resistance compared with long-term response [21]. Huang et al. [22] have found that serum miR-376a/b/c is significantly downregulated in glioma patients compared with the healthy controls, proposing a potential role of serum miR-376a/b/c as noninvasive biomarkers for diagnosis and prognosis in glioma. Our data also showed that the expression of serum exosomal miR-376b was decreased in both intracranial lymphoma and high-grade glioma, indicating a potential role for disease diagnosis.

Collectively, we showed that miR-766-5p and miR-376b-5p in serum exosomes might be used as auxiliary diagnostic indicators for high-grade glioma and intracranial lymphoma, and miR-766-5p might be used as a differential diagnostic marker for both diseases, providing a new noninvasive marker for disease detection.

## Figures and Tables

**Table 1:** Demographic and clinical characteristics of patients.

	High-grade glioma (*n* = 32)	Intracranial lymphoma (*n* = 12)
Age, median (25%-75%)	43 (26-49)	47 (22-53)
Gender		
Male	20	8
Female	12	4
Pathological pattern	Anaplastic astrocytoma 15	Diffuse large B cell lymphoma 12
	Glioblastoma 17	
WHO classification		
III	15	
IV	17	
P53-positive cells (%)	25% (25%-50%)	10% (0%-25%)
Ki67-positive cells (%)	35% (20%-60%)	75% (50%-90%)

## Data Availability

The data used to support the findings of this study are available from the corresponding author upon request.

## Abbreviations

ROC: Receiver operating characteristic
AUCs: Areas under ROC curve
miRNA: MicroRNA
KEGG: Kyoto Encyclopedia of Genes and Genomes.
